# Phylogenetic distribution and experimental characterization of corrinoid production and dependence in soil bacterial isolates

**DOI:** 10.1093/ismejo/wrae068

**Published:** 2024-04-22

**Authors:** Zoila I Alvarez-Aponte, Alekhya M Govindaraju, Zachary F Hallberg, Alexa M Nicolas, Myka A Green, Kenny C Mok, Citlali Fonseca-García, Devin Coleman-Derr, Eoin L Brodie, Hans K Carlson, Michiko E Taga

**Affiliations:** Department of Plant & Microbial Biology, University of California, Berkeley, Berkeley, CA 94720, United States; Department of Plant & Microbial Biology, University of California, Berkeley, Berkeley, CA 94720, United States; Department of Plant & Microbial Biology, University of California, Berkeley, Berkeley, CA 94720, United States; Department of Plant & Microbial Biology, University of California, Berkeley, Berkeley, CA 94720, United States; Department of Plant & Microbial Biology, University of California, Berkeley, Berkeley, CA 94720, United States; Department of Plant & Microbial Biology, University of California, Berkeley, Berkeley, CA 94720, United States; Department of Plant & Microbial Biology, University of California, Berkeley, Berkeley, CA 94720, United States; Plant Gene Expression Center, USDA-ARS, Albany, CA 94710, United States; Department of Plant & Microbial Biology, University of California, Berkeley, Berkeley, CA 94720, United States; Plant Gene Expression Center, USDA-ARS, Albany, CA 94710, United States; Climate and Ecosystem Sciences Division, Lawrence Berkeley National Laboratory, Berkeley, CA 94720, United States; Department of Environmental Science, Policy and Management, University of California, Berkeley, Berkeley, CA 94720, United States; Climate and Ecosystem Sciences Division, Lawrence Berkeley National Laboratory, Berkeley, CA 94720, United States; Department of Plant & Microbial Biology, University of California, Berkeley, Berkeley, CA 94720, United States

**Keywords:** soil microbiome, bacteria, isolation, vitamin B12, corrinoid, cobamide, genomic predictions, metabolic interactions, model nutrients

## Abstract

Soil microbial communities impact carbon sequestration and release, biogeochemical cycling, and agricultural yields. These global effects rely on metabolic interactions that modulate community composition and function. However, the physicochemical and taxonomic complexity of soil and the scarcity of available isolates for phenotypic testing are significant barriers to studying soil microbial interactions. Corrinoids—the vitamin B_12_ family of cofactors—are critical for microbial metabolism, yet they are synthesized by only a subset of microbiome members. Here, we evaluated corrinoid production and dependence in soil bacteria as a model to investigate the ecological roles of microorganisms involved in metabolic interactions. We isolated and characterized a taxonomically diverse collection of 161 soil bacteria from a single study site. Most corrinoid-dependent bacteria in the collection prefer B_12_ over other corrinoids, while all tested producers synthesize B_12_, indicating metabolic compatibility between producers and dependents in the collection. Furthermore, a subset of producers release B_12_ at levels sufficient to support dependent isolates in laboratory culture at estimated ratios of up to 1000 dependents per producer. Within our isolate collection, we did not find strong phylogenetic patterns in corrinoid production or dependence. Upon investigating trends in the phylogenetic dispersion of corrinoid metabolism categories across sequenced bacteria from various environments, we found that these traits are conserved in 47 out of 85 genera. Together, these phenotypic and genomic results provide evidence for corrinoid-based metabolic interactions among bacteria and provide a framework for the study of nutrient-sharing ecological interactions in microbial communities.

## Introduction

Microorganisms engage in metabolic interactions that collectively define ecological networks in communities [1]. Microbial interactions can be key mediators of community function, and disruptions to interactions can restructure whole communities [2, 3]. Thus, it is crucial to disentangle microbial interactions and generate a predictive understanding of nutritional influences on communities and, in turn, on the environment.

Experimental and computational studies have shown that microorganisms commonly lack the ability to synthesize all of the metabolites they require [4, 5]. For example, many microorganisms are unable to synthesize certain cofactors and amino acids, and therefore must acquire these nutrients from other organisms in the environment [6–8]. As a consequence, microbial communities are composed of “producer” and “dependent” organisms that synthesize and require a given nutrient, respectively. The complexity of microbial communities is due, in part, to this network of interactions arising from interdependence among microorganisms that produce and require a range of different nutrients. Such interdependence may develop because loss of biosynthesis genes can be evolutionarily favored in contexts where required nutrients are abundant in the environment or can be acquired from other microorganisms [9, 10].

Investigating the molecular mechanisms and community impacts of nutritional interactions is challenging for many reasons. First, many microbial communities are functionally diverse and contain numerous metabolites that are produced, used, and chemically transformed by community members. Second, although some metabolic capabilities can be inferred from genomic data, these analyses currently lack spatial and temporal resolution, making it difficult to predict how interactions among community members may be impacted by the metabolic activities of a single microbe. Third, because most microorganisms have not been isolated in pure culture [11], metabolic predictions of the uncultured majority have yet to be confirmed. The challenges common to microbiome studies are amplified in soil due to its taxonomic diversity, physical and chemical heterogeneity, and environmental fluctuations, as well as disturbances due to animal or human activities [12–14]. Nonetheless, generating mechanistic knowledge of nutrient cycling in soil communities is essential because of their broad impacts on the health of our planet [15, 16]. We address these challenges by studying the ecological roles of microorganisms in relation to one class of model shared nutrients in a collection of newly isolated soil bacteria.

**Figure 1:**
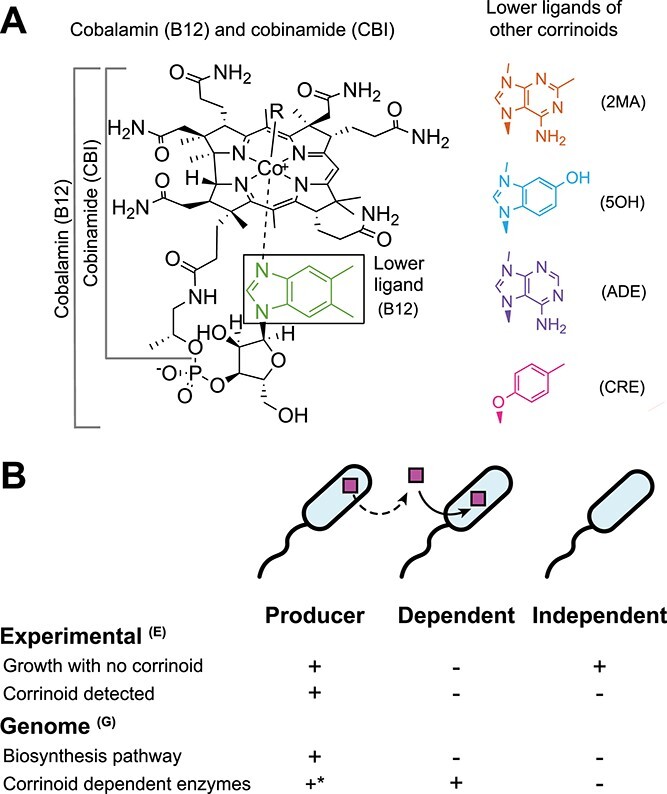
Diversity of corrinoid lower ligands and metabolic roles; (A) chemical structure of cobalamin (B12), lower ligands of other corrinoids used in this research, and three-letter abbreviations; from the top, 2MA, 5OH, ADE, and CRE; CBI, shown on the left, is an incomplete corrinoid that does not contain a lower ligand; (B) corrinoid metabolism categories include producers, dependents, and independents; producers may release corrinoids (dashed line); these categories can be assigned based on experimental results, denoted with superscript E, and genomic analysis, denoted with superscript G, as summarized in the table; ^*^in principle, producers^G^ are defined based solely on the presence and completeness of the biosynthesis pathway, but all corrinoid producer^G^ genomes examined thus far encode one or more corrinoid-dependent enzymes [25].

We took a reductionist approach to investigate nutrient production and dependence by focusing on corrinoids as a representative class of shared metabolites. Corrinoids are produced by a subset of bacteria and archaea and include the vitamin B_12_ (cobalamin) family of cobalt-containing cobamide cofactors and their biosynthetic precursors (Fig. 1A). Corrinoids are required cofactors for methionine synthesis and propionate metabolism in most eukaryotes, and additionally are used by prokaryotes for other diverse processes such as mercury methylation, natural product biosynthesis, nucleotide synthesis, and numerous carbon and nitrogen transformations [17]. Corrinoid-sharing interactions between producer and dependent microorganisms have been observed in laboratory co-cultures of bacteria [18–20], bacteria-microeukaryote pairs [21–23], and in higher order consortia [24], and are thought to be prevalent in the gut microbiome [7].

Computational predictions further support the hypothesis that corrinoids are broadly shared, as 37% of sequenced bacterial species are predicted corrinoid producers and 49% are dependents [25]. The remaining 14% are predicted not to produce or use corrinoids, fulfilling their metabolic needs via corrinoid-independent pathways, and are therefore considered “independents” (Fig. 1B) [25]. Given that dependents and producers coexist in the same environments [26–28], we hypothesize that many sharing interactions have yet to be described.

An aspect of corrinoids that influences their function as shared nutrients is their structural diversity (Fig. 1A), which has been shown to impact function: microbial preferences for particular corrinoid structures are apparent in their differential growth responses to corrinoids [17, 29]. Distinct groups of corrinoids have been detected in a variety of host-associated and environmental microbial communities, suggesting that microorganisms encounter diverse corrinoids in nature [30]. Because most corrinoids are not commercially available, nearly all research on corrinoids has been performed only with B_12_ [17]. Given that some microorganisms require corrinoids other than B_12_ [31], it is likely that novel bacteria that could not have been isolated on B_12_ will be culturable on other corrinoids.

In this study, we investigated the impact of corrinoid structure on bacterial growth and isolation from a California annual grassland soil. We generated a diverse collection of soil bacterial isolates and experimentally confirmed that all three predicted corrinoid metabolism categories—producer, dependent, and independent—are represented in the soil microbial community. Further, the structure and amount of corrinoid released by some producers are compatible with the requirements of corrinoid-dependent isolates, suggesting that corrinoid sharing can occur between these bacteria in soil. A broad analysis of bacterial genomes revealed that it is possible to classify some bacterial taxa as corrinoid producers, dependents, or independents based on phylogeny alone. Our results provide an ecological framework for understanding nutritional interactions in soil through the lens of corrinoids.

## Materials and Methods

### Isolation of bacteria from soil by the limiting dilution method

We collected soil samples from top 10 cm of an annual grassland at the Hopland Research and Extension Center in Hopland, CA (39.004056 N 123.085861 W) in November 2019 and again in April 2021 to increase the total number of isolates. Site characteristics and soil physicochemical properties for our sampling site were previously documented [32, 33]. Soil pH, determined by preparing a slurry with one part soil to two parts deionized water and measuring (*n* = 3) with an Orion Star A111 pH Meter (Thermo Scientific, Waltham, MA), was 5.87 ± 0.42 in November 2019 and 6.28 ± 0.07 in April 2021.

To separate microbial biomass from the soil, we resuspended 2.5 g of soil in 25 ml phosphate-buffered saline with 2.24 mM sodium pyrophosphate (Alfa Aesar, Heysham, England), stirred for 30 min, and allowed the slurry to settle for 15 min before diluting the supernatant. All isolations and subsequent growth steps were performed using a modified VL60 medium (pH 6.0) [34] (Supplementary Table 1), amended with 0.1 g/l each of xylose, xylan, N-acetylglucosamine, and glucose, as well as 10 nM B_12_ (B12), 2-methyladeninylcobamide (2MA), 5-hydroxybenzimidazolylcobamide (5OH), adeninylcobamide (ADE), *p*-cresolylcobamide (CRE), or cobinamide (CBI) when indicated. Cultures were grown at room temperature unless otherwise noted. The corrinoids 2MA, 5OH, and ADE were produced by guided biosynthesis in *Propionibacterium acidipropionici* and CRE in *Sporomusa ovata*; extracted from bacterial cultures; and purified as previously described [35, 36]. We conducted a most probable number (MPN) count to determine the soil slurry dilution required to reach growth in ~30% of wells, a density that is expected to yield 80% clonal cultures, based on a Poisson distribution and previously reported isolations from human stool samples [37]. About 80 μl aliquots of the soil solution diluted in each medium were dispensed into the wells of 384-well plates using a Biomek liquid handler (Beckman Coulter, Indianapolis, IN). Three plates per corrinoid condition were inoculated, and an uninoculated plate was prepared for each condition, for a total of 28 plates. Plates were covered with BreatheEasy (Diversified Biotech, Dedham, MA) membranes for this and all subsequent steps, and incubated statically at room temperature for 44 days. Despite using the MPN calculation to determine the dilution, a surprisingly low number of wells showed growth in the isolation from soil collected in November 2019 (2.4%). Cultures from wells in which the OD_600_ (measured on a Tecan Spark plate reader (Grödig, Austria)) exceeded 0.29 were transferred into fresh medium at the end of the initial incubation and grown for up to 40 days. The cultures were then split into two portions, one stored at −80°C in 25% glycerol and another prepared for sequencing. The full 16S rRNA gene was amplified by polymerase chain reaction (PCR) from each well with primers 27F and 1492R [38] (IDT, Coralville, IA) and Dream*Taq* polymerase (Thermo Scientific). PCR purification and Sanger sequencing of all amplicons using the same primers were done at the UC Berkeley DNA Sequencing Facility. Sanger sequence trimming with a 0.01 error probability cutoff and de novo assembly of reads were performed on Geneious Prime (2022.1.1). Cultures with a single, high-quality 16S rRNA gene sequence were considered clonal.

Prior to the second isolation from soil collected in April 2021, the soil sample was stored at 4°C for 1 month, brought to 20% moisture from an original 3.33 ± 0.58% with sterile deionized water, and incubated for 1 week. After diluting the soil and dispensing into 96-well plates, the plates were incubated at room temperature for 49 days. The percentage of wells showing growth was much higher than in the previous isolation round (37% of total wells). Therefore, high-throughput sequencing of the 16S rRNA gene V4/V5 region was performed as previously described [39] with 600 bp v3 reagents. This step allowed us to select wells containing a single amplicon sequence variant, which were assumed to be clonal cultures. To determine the total phyla represented across all wells, 16S rRNA gene sequences were searched against the RDP database (release 8.0) [40] (Supplementary Fig. 1).

Liquid cultures prepared from glycerol stocks were purified by streaking on 2X modified VL60 solidified with 14 g/l Difco noble agar (BD, Sparks, MD). Nystatin (63 ng/ml) was added to the medium in cases where fungal contamination was observed. Cultures were serially purified by streaking until all observed colonies were of uniform morphology. For each isolate, liquid cultures inoculated from a single colony were stored at −80°C in 40% glycerol. After purifying, the identity of each isolate was confirmed by a second round of Sanger sequencing.

We identified 23 sequences with higher than 99% pairwise identity to a sequence in an uninoculated well. These were considered potential contaminants and removed from the dataset. After removal of isolates with chimeric sequences, the final collection is composed of 161 isolates.

Growth curves were generated to classify isolates into groups based on the time they required to reach saturating growth (24, 48, 168, or 336 h). Isolates were inoculated from glycerol stocks into 96-well plates in triplicate and grown for 168 h at 28°C, shaking at 800 rpm in a plate shaker (Southwest Science, Roebling, NJ), and separately at room temperature with no shaking. Growth curves were generated by measuring OD_600_ at 0, 6, 12, 24, 36, and 48 h, and every 24 h until 168 h for the shaken cultures and 216 h for standing cultures. Because isolates grew more consistently in the shaking condition, cultures were shaken at 28°C for all subsequent steps.

### Experimental characterization of isolates as corrinoid producers, dependents, or independents

To determine whether isolates were dependent on corrinoids for growth, isolates in the 24-, 48-, and 168-h groups were inoculated into 96-well plates with 200 μl of media containing the corrinoid used for isolation. Following growth to saturation, each culture was diluted into two wells, one amended with the same corrinoid and the other with no corrinoid (NOC), using a multi-blot replicator that transferred ~3 μl per well (V&P Scientific, San Diego, CA). Cultures were serially passaged three additional times into the same media to eliminate corrinoid carryover. OD_600_ was measured before and after each passage. Isolates that did not grow reproducibly in media with corrinoid were not pursued further (24 isolates). Isolates that continued to grow in media with corrinoid but stopped growing after being transferred into media with NOC were classified as dependents^E^, while those that continued to grow in both conditions were considered to be either producers^E^ or independents^E^ (Fig. 1B). Superscript E is used to distinguish experimental results from genomic predictions (superscript G, discussed below). To evaluate the effect of corrinoids on the growth of isolates, we calculated the corrinoid-specific growth enhancement as log_2_ [1 + ((OD_with corrinoid_ – OD_no corrinoid_)/(OD_no corrinoid_))] [41] and determined a threshold for corrinoid dependence based on the growth of bacteria isolated in the NOC condition that also underwent serial transfer (maximum value obtained from the equation plus standard deviation). If two or three of the three replicates were classified as corrinoid dependent, corrinoid dose–response assays were performed to confirm dependence and determine the corrinoid preferences of each isolate. For dose–response curves, isolates were precultured in media with 10 nM corrinoid, followed by a second preculture step in media with NOC, and inoculated into 12 different concentrations of each corrinoid (ranging from 10 nM to 2.4 fM). Final ODs were measured and curves were fitted using a four-parameter non-linear fit on GraphPad Prism (v9.5.1) to determine each EC_50_.

To distinguish producers^E^ from independents^E^ (Fig. 1B), 100 μl of each culture were collected at the end of the fourth passage into NOC medium and lysed by incubating at 98°C for 20 min. An *Escherichia coli*-based corrinoid detection bioassay was conducted as previously described [35] to determine the presence or absence of corrinoid in each sample. Data were processed to yield a “growth due to corrinoid” metric by subtracting growth due to methionine (as measured by the ∆*metE*∆*metH* control strain) from growth of the ∆*metE* bioassay strain and normalizing to growth of the wildtype *E. coli* strain. An isolate was characterized as a producer^E^ if the normalized result was greater than or equal to 2 or if the OD_600_ of the ∆*metE* bioassay strain was greater than or equal to 0.1. Conversely, an isolate was characterized as a non-producer, and thus an independent^E^, if the normalized result was <2 and the *E. coli* ∆*metE* OD_600_ was <0.1. Our method was validated using a set of previously isolated soil bacteria [42] that were genomically predicted to be corrinoid producers (Supplementary Table 2). Isolates that repressed growth of *E. coli* (2 isolates), grew to an OD_600_ <0.1 (7 isolates), or for which the three replicates or results for the dependence and production were inconsistent (11 isolates) were deemed inconclusive.

Data processing and analysis were performed using Python 3.7 on Jupyter Notebooks (version 6.2.0) and all code is on GitHub under DOI: 10.5281/zenodo.10815172.

For further characterization of producers, we grew 1 l cultures of each in VL60 medium with no corrinoid and 200× amino acids and extracted corrinoids as described previously [35]. Corrinoid extracts were analyzed by high-pressure liquid chromatography (HPLC) on a 1200 series HPLC system equipped with a diode array detector (Agilent Technologies, CA) and compared to authentic corrinoid standards using Method 2, as described previously [31].

### Genus-based predictions of corrinoid metabolism

We used a previously developed dataset [25], which reports predictions of corrinoid biosynthesis and dependence for 11 436 bacterial species, and used these existing predictions to further genomically characterize bacterial species into the producer^G^, dependent^G^, and independent^G^ categories. Species that were previously classified as very likely, likely, or possible producers were considered producers^G^ [25]. Corrinoid-dependent^G^ species were defined as those previously classified as very likely or likely non-producers that also had at least one corrinoid-dependent function, regardless of whether their genomes encoded specific corrinoid-independent alternative enzymes [25]. Corrinoid-independent^G^ species were defined as those that were likely or very likely non-producers and had no corrinoid-dependent functions. After classifying each species, we grouped all species into their respective genera (JGI IMG taxonomic metadata was downloaded on 18 July 2023, to update any reclassified genomes). To establish a reliable cutoff for our predictions, we chose genera containing 20 species or more and made a genomic classification when 95% or more of the species in a genus corresponded to the same category.

### Phylogenetic tree building

The phylogenetic tree of the isolates was constructed from full-length 16S rRNA gene sequences (Fig. 3). Isolate taxonomy assignment and tree building were done using the Silva Alignment, Classification, and Tree service [43]. To determine whether isolates were likely novel, we used BLAST to search assembled sequences against the NCBI Reference Database using Geneious (2022.1.1). If the pairwise identity between the isolate sequence and the top hit was lower than 98.6%, we considered the isolate to be novel [44].

**Figure 2:**
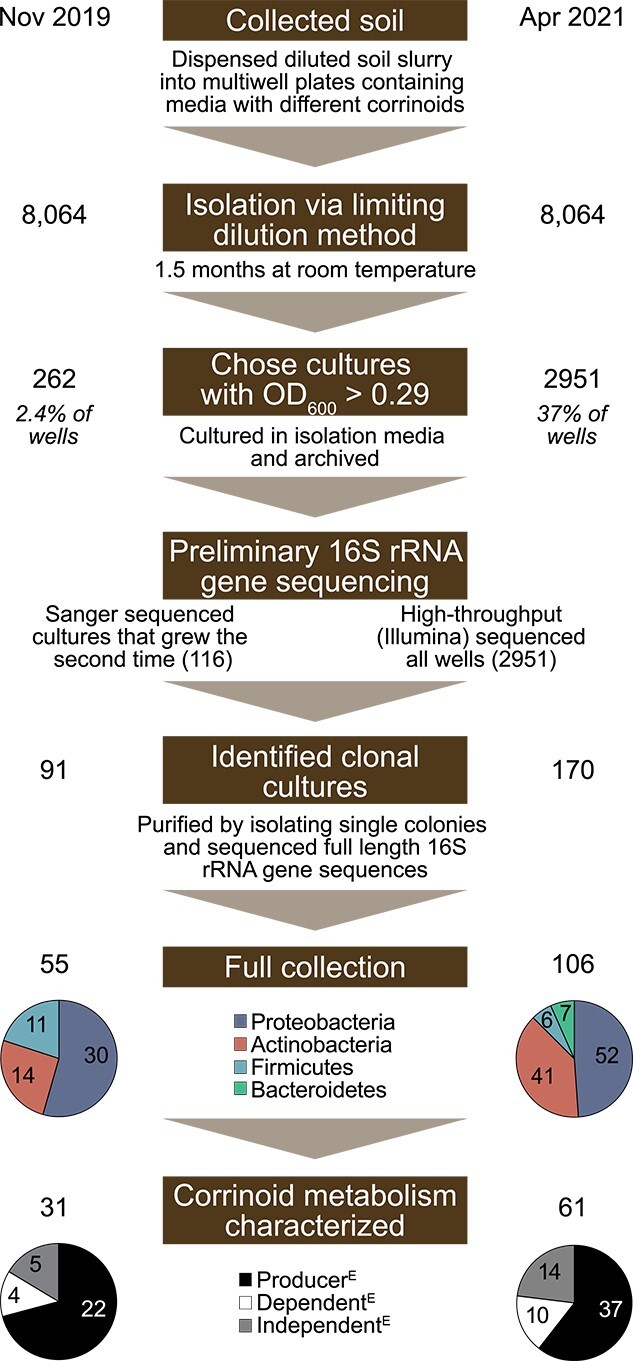
Overview of the experimental procedure; soil bacteria were isolated by the limiting dilution method on media containing different corrinoids; growth was detected based on OD_600_, and clonal isolates were distinguished from mixed cultures by 16S rRNA gene sequencing; after several purification steps, the final isolate collection contains 161 clonal isolates, of which 92 were successfully characterized as corrinoid producer^E^, dependent^E^, or independent^E^.

**Figure 3:**
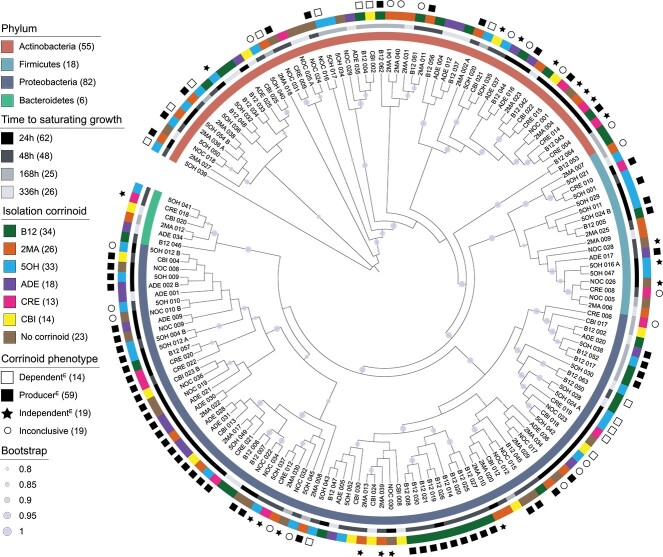
Overview of the isolate collection; a phylogenetic tree was built from the full-length 16S rRNA gene sequences of the isolates; the six- to seven-character ID is shown for each isolate; circles on tree branches show bootstrap values above 0.8; rings from the inside out show, for each isolate, (i) phylum as identified by SILVA taxonomy, (ii) time to saturating growth determined from growth curves, (iii) the corrinoid used in the isolation medium, and (iv) the experimentally determined corrinoid metabolism category; isolates with no symbol on the fourth ring were not tested due to slow or inconsistent growth; numbers in parentheses correspond to the number of isolates belonging to each category.

**Figure 4:**
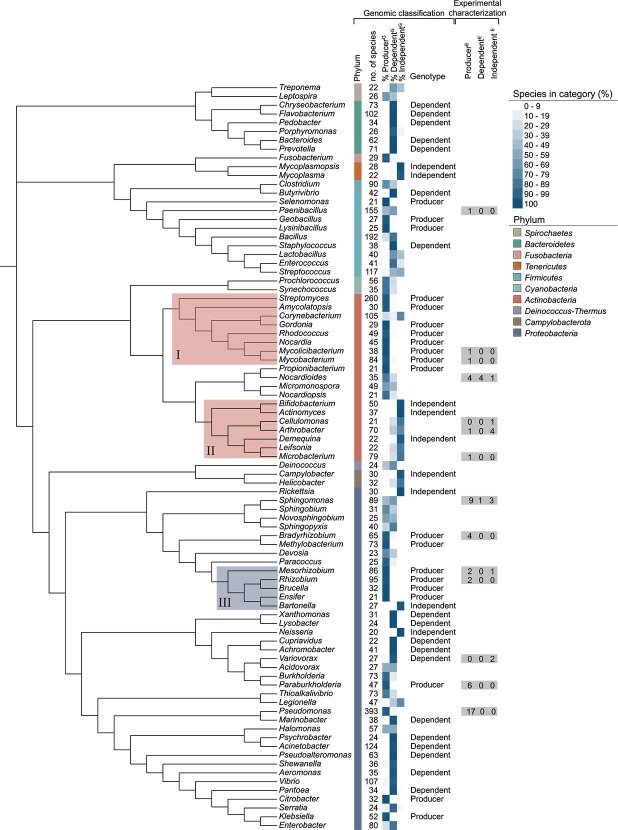
Genome-based predictions of corrinoid metabolism at the genus level; the phylogenetic tree was built from the full-length 16S rRNA gene sequences of the type species of 85 genera from the dataset in Shelton *et al.* [25] that met our cutoff by having 20 species or more; the first two columns show the phylum and the number of species analyzed for each genus, respectively, which total 10 phyla and 4720 species; the next three columns show the percent of species in each genus predicted to belong to each corrinoid metabolism category; a corrinoid-specific genotype is indicated if 95% or more species in a genus belong to the same category; the columns labeled experimental characterization show the number of isolates in the collection found to belong to each category based on experimental results; shaded regions I, II, and III denote clades discussed in the text; the unpruned tree that was used to generate the figure is shown in Supplementary Fig. 3, and sequences obtained from the NCBI Reference Sequence database can be found in Supplementary Table 3.

The genus level phylogenetic tree was generated using the full-length 16S rRNA gene sequences for the type species of each genus (85 species) and 35 additional type species that were added for context and later pruned (Fig. 4, Supplementary Table 3, Supplementary Fig. 3). A MUSCLE [45] alignment and FastTree [46] were used to generate the tree on Geneious (2022.1.1) using default settings. Tree pruning and annotation for both trees were performed on iTOL [47].

## Results

### Generating a collection of 161 bacterial isolates from soil

To generate a collection of soil bacterial isolates with a diversity of corrinoid requirements, we performed the limiting dilution method [37] in 384-well plates containing media with one of six different corrinoids (Fig. 1A) or no corrinoid. The isolation was performed in two rounds (Fig. 2 November 2019 and April 2021). In the first isolation, 8064 inoculated wells yielded only 2.4% of wells with detectable growth. 16S rRNA gene sequencing of the resulting cultures revealed that 78% were clonal. In the second isolation round, 8064 wells were inoculated and 37% contained detectable growth, yet only 5.8% of cultures were clonal. By statistical metrics alone, we expected 80% of cultures to be clonal when 30% of wells contained detectable growth. These results suggest that smaller soil inocula lead to more clonal cultures, possibly because cell aggregation is more prevalent in soil than in the gut environment, where the same method led to the statistically predicted result [37]. Twenty phyla were found in the total collection (Supplementary Fig. 1), but after selecting clonal cultures, reviving them from frozen stocks, purifying, and archiving, our collection contained 161 bacterial isolates, representing four phyla, which were used for subsequent analyses (Fig. 2).

The isolate collection is dominated by the phyla *Proteobacteria* and *Actinobacteria*, with fewer representatives from the *Firmicutes* and *Bacteroidetes* phyla (Fig. 3). This is similar to the relative abundances observed in bulk soil, where *Proteobacteria* and *Actinobacteria* are the dominant phyla [33, 48, 49]. Of the 161 isolates, 23% (37 isolates) were considered to be novel species [44]. The collection comprises 31 genera and 121 unique 16S rRNA gene sequences, with 11 genera each represented by a single isolate and three genera represented by 18 or more isolates. Despite this diversity, we have not sampled the bacterial diversity in this soil exhaustively (Supplementary Fig. 2).

### Taxonomic and phenotypic characterization of the isolate collection

To investigate whether there were phylogenetic trends for the observed phenotypes, we constructed a phylogenetic tree of the isolate collection annotated with the characteristics of each isolate (Fig. 3, Supplementary Table 4). We did not observe strong phylogenetic trends in the time required for each isolate to reach saturating growth, except that some clades of *Proteobacteria* contained only fast-growing isolates. Similarly, we did not observe a correlation between phylogeny and the corrinoid used for isolation. An exception was a clade of producers within *Proteobacteria*, all *Sphingomonas*, that were isolated on B12. The number of isolates recovered in B12, 5OH, and 2MA was higher than the number of isolates in the NOC condition, while ADE, CBI, and CRE led to the recovery of fewer isolates than NOC (Fig. 3).

### Classifying corrinoid metabolism phenotypes in the isolate collection

We experimentally classified each isolate as a corrinoid producer, dependent, or independent. We first assessed growth in the presence and absence of corrinoid. Isolates that stopped growing following serial transfer into media without corrinoid were classified as dependents^E^. Isolates that could grow in the absence of corrinoid were tested for corrinoid production using an *E. coli-*based corrinoid detection bioassay [35] to distinguish producers^E^ from independents^E^ (Fig. 1B; see Materials and Methods). Based on these results, all three categories are represented in the isolate collection, with the majority (64%) classified as producers^E^, and 14 (15%) classified as dependents^E^ (Fig. 3). The abundance of producers^E^ in our collection contrasts with genome-based predictions that dependents outnumber producers across bacteria and specifically in soil [25, 26, 50].

To investigate potential phylogenetic trends in corrinoid metabolism categories, we overlaid the experimentally determined corrinoid phenotypes onto the phylogenetic tree (Fig. 3). At the phylum level, we observed mixed phenotypes. For example, all three categories are represented among characterized *Actinobacteria* and are interspersed across several clades. In contrast, in the *Proteobacteria*, the corrinoid phenotypes are largely consistent with phylogeny. Most *Proteobacteria* clades are composed of only producer isolates, aside from one clade containing genera *Phenylobacterium* and *Caulobacter* that is composed of only dependents, while in a few other clades, the phenotypes are interspersed. Thus, although trends are seen in some closely related isolates, large-scale phylogenetic trends in corrinoid phenotype are not apparent.

### Corrinoid metabolism is conserved in a subset of genera, enabling taxonomy-based metabolic predictions

Although we did not observe strong phylogenetic trends for corrinoid metabolic categories in our isolate collection, we hypothesized that trends might exist across the bacterial domain at large. The existence of strong phylogenetic patterns for corrinoid metabolic categories would enable the prediction of corrinoid metabolism based solely on phylogeny, and having an isolate collection would allow us to confirm these predictions. We analyzed our previously published corrinoid metabolism classifications for over 11 000 bacterial species [25] to distinguish between the competing hypotheses that (i) corrinoid production, dependence, and independence show strong phylogenetic trends, enabling predictions of corrinoid metabolism based on taxonomy, or (ii) corrinoid metabolism categories are phylogenetically interspersed, making it impossible to infer corrinoid-related ecological roles based solely on taxonomy. In our previous study, trends were not apparent at the phylum level except in the *Bacteroidetes*, which were nearly all dependents^G^ [25]. Therefore, we aimed to evaluate trends at lower taxonomic levels, starting with the genus level.

We searched for phylogenetic trends among the 85 genera in our dataset and classified each genus as producer^G^, dependent^G^, or independent^G^ when possible. A corrinoid metabolism category could be assigned with high confidence for 47 out of 85 genera (Fig. 4, Supplementary Table 5). In the remaining 38 genera, a single corrinoid metabolism category does not predominate, so corrinoid metabolism classifications could not be made.

To evaluate trends across higher taxonomic levels, we mapped the genomic predictions onto a phylogenetic tree constructed from the full 16S rRNA gene sequences of the type species for each genus (Fig. 4, Supplementary Fig. 3). As expected, five of the six *Bacteroidetes* genera were predicted to be dependent^G^ and the sixth *Bacteroidetes* genus (*Porphyromonas*) has a small percentage of independent^G^ species. We observed phylogenetic trends at levels higher than the genus level in some cases. The *Actinobacteria* form two distinct clades, one dominated by producers^G^ (Clade I in Fig. 4) and the other by independents^G^ (Clade II). No *Actinobacteria* genera were classified as dependents^G^, although some genera have low percentages of dependent species. All genera in one *Proteobacteria* clade are classified as producers^G^ (Clade III), with the notable exception of *Bartonella*, which has undergone genome reduction [51] and is classified as independent^G^. However, other *Proteobacteria* clades were mixed, aside from a few sister taxa that share corrinoid genotypes in some instances, such as *Bradyrhizobium and Methylobacterium* which are both producers^G^ and *Xanthomonas* and *Lysobacter*, which are both dependents^G^. For phyla that had fewer genera, the few classified genera were independent^G^. This may be due to a bias in the dataset, which is composed of over 90% cultured bacteria. Because cultured bacteria tend to have larger genomes and fewer auxotrophies than uncultured bacteria [4], they are less likely to be corrinoid-dependent. Future analysis of metagenomes may reveal more dependence among phyla with fewer sequenced representatives.

Upon comparing the genomic classifications to our experimental results for the isolate collection, we found that 19 isolates belong to genera for which genomic classifications were possible. All isolates except one matched the genomic classification (Fig. 4). The exception was a *Mesorhizobium* isolate that was predicted to be a producer^G^ but found to be independent^E^, suggesting either that it is not capable of synthesizing a corrinoid or did not produce a detectable amount under our growth conditions. The confirmation of our genomic classifications with experimental data from our isolate collection lends support to the phylogenetic predictions made for certain genera.

### Corrinoid preferences of dependent isolates reveal diverse corrinoid use capabilities

**Figure 5:**
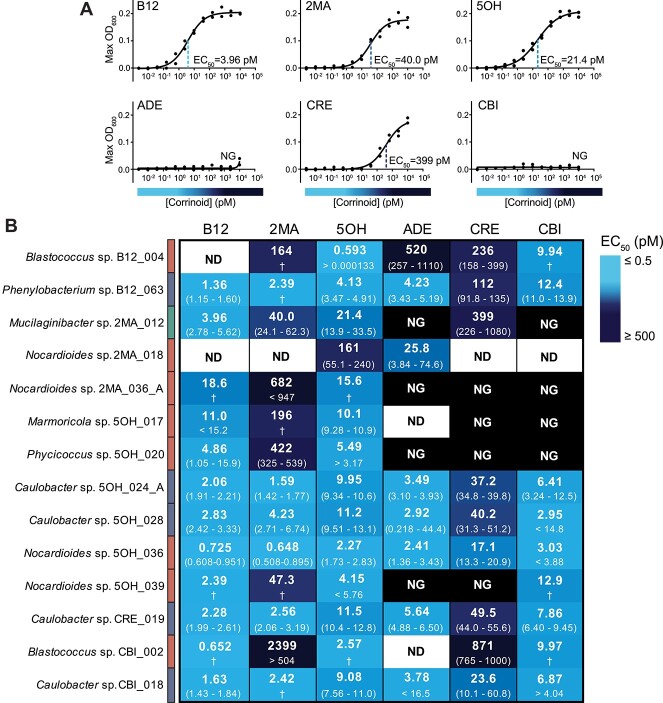
Corrinoid dependence in the isolate collection; (A) representative dose–response curves belonging to isolate 2MA_012; lines show non-linear fit for each corrinoid (*n* = 2); dotted line marks the EC_50_ for each corrinoid and the EC_50_ is shown when one was obtained; (B) the corrinoid concentrations resulting in half-maximal growth (EC_50_) are shown for all 14 corrinoid-dependent isolates on the six corrinoids used in this study; for each isolate and corrinoid combination, the top number is the EC_50_, and the numbers in parentheses represent the 95% confidence interval as calculated by a four-parameter non-linear fit on GraphPad Prism (v9.5.1); greater than and less than symbols were used when the upper or lower bound of the confidence interval could not be determined, respectively; the phylum each isolate belongs to is shown in the bar next to the isolate names, colors correspond to the legend in Figs 2 and 3; NG, no growth; ND, no data when a regression was not possible due to poor data quality, † 95% confidence interval not determined; curve fit data are summarized in Supplementary Table 7.

Our observation that the corrinoid used for isolation did not correlate with phylogeny (Fig. 3) led us to investigate the corrinoid preferences of the 14 dependent isolates in our collection. We measured growth in media containing a range of concentrations of different corrinoids and calculated the corrinoid concentration that resulted in half-maximal growth (EC_50_); the corrinoid with the lowest EC_50_ is considered the most preferred (Fig. 5A and Supplementary Fig. 4). The corrinoid used for isolation was not the most preferred corrinoid in many cases, likely because the corrinoid concentration in the isolation medium was in 100- to 1000-fold excess of isolate requirements (Fig. 5B). Further, despite our previous finding that most corrinoids other than B12 have not been detected in this soil [30], we found that all of the isolates can use at least one corrinoid in addition to B12, with B12 preferred by almost all isolates. ADE, CRE, and CBI could not be used at any of the tested concentrations by some isolates. These were the same corrinoids in which we recovered the lowest numbers of isolates, suggesting that only some isolates can use the complete corrinoids ADE and CRE as cofactors or salvage CBI to make a complete corrinoid under our experimental conditions [52–54].

### B12 is the main corrinoid produced by isolates in the collection and it is only provided by a subset of producers

**Figure 6:**
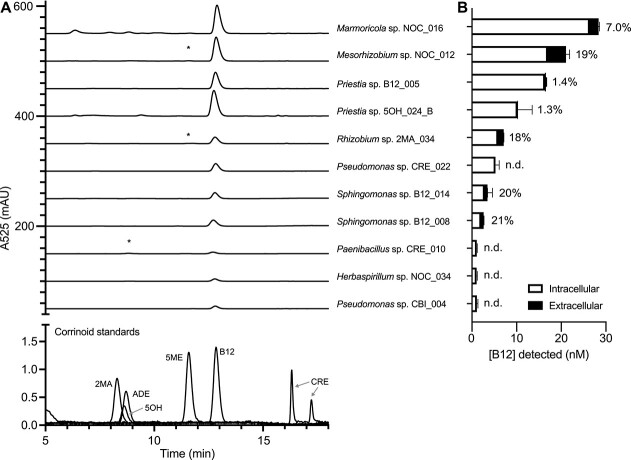
Corrinoid production and providing in the isolate collection; (A) HPLC analysis of corrinoid extracts of 11 selected producers shows B12 is the major corrinoid produced; authentic corrinoid standards are shown at the bottom; asterisks denote small peaks that indicate the presence of a second complete corrinoid; (B) quantification of corrinoids in the cell pellet (intracellular) and supernatant (extracellular) fractions of each isolate as detected by an *E. coli*-based corrinoid bioassay; the percent of corrinoid provided (extracellular corrinoid as a fraction of the total corrinoid) is given to the right of each bar; bars and error bars show the average and standard deviation of three technical replicates, respectively; n.d., extracellular corrinoid was not detected.

Given that dependents need to obtain corrinoids from producers in their community, we sought to determine whether there is compatibility between the corrinoids produced and required by isolates in our collection. To that end, we extracted corrinoids from cultures of 12 fast-growing producers and analyzed them by HPLC. We detected a corrinoid by HPLC in 11 of these producers. The other isolate did not show a signal when re-tested with the *E. coli* bioassay and was reclassified as inconclusive. Comparison with authentic standards revealed that B12 was the dominant corrinoid synthesized by the 11 tested producers (Fig. 6A).

Although it is unknown to what extent dependents acquire corrinoids directly from producer cells, a recent report showed that some producers cultured in the laboratory can release corrinoids into the growth medium [23]. We used the *E. coli*-based bioassay to quantify the corrinoids in culture supernatants and cell pellets of the 11 producer isolates. Seven of the producers were found to be “providers” [23]—producers for which corrinoids were present in the culture supernatant—while corrinoids were detected exclusively in the cell pellet fraction in the remaining four producers (Fig. 6B). The amount of provided corrinoid ranged from 1.3% to 21% of the total corrinoid, and the provided amount did not correlate with the total amount of corrinoid produced. The concentrations of provided corrinoid are 1 to 1000 times higher than the EC_50_ values calculated for the dependents (Fig. 5B, Supplementary Table 6), suggesting that these isolates have the capacity to provide sufficient or excess corrinoid to all of the dependents in our collection. Thus, our measurements of corrinoid production and providing, in the artificial conditions of laboratory culture, coupled with prior genomic studies [25, 26], support our hypothesis that corrinoid sharing can occur within the communities of this soil.

## Discussion

Microbial nutritional interactions play pivotal roles in establishing community structure and function. Characterizing and predicting the ecological roles of microorganisms as nutrient producers and dependents can contribute to the understanding of microbial interaction networks and their influence on the whole community. Here, we investigated the ecological roles of microorganisms by overlaying experimental and computational approaches. We were able to characterize specific functional roles of bacteria by focusing on a single class of model nutrients, corrinoids, the sharing of which is thought to be widespread in microbial communities [17, 25, 27, 28]. The importance of corrinoids for soil bacteria has long been recognized [50, 55–57]. Here, we report the first systematic isolation and characterization of soil bacteria on corrinoids other than B12, allowing us to consider the ecological roles of corrinoid producers and dependents in the context of soil microbial ecology.

Microorganisms typically have preferences for different corrinoids that are reflected in their EC_50_ values [17, 29, 58]. These preferences result from corrinoid transport efficiency, the affinity of corrinoids for the enzymes that use them, and what corrinoid dependent processes are in use [58–60]. Corrinoid preferences, combined with the availability of corrinoids in a given environment and competition for corrinoids in the community, can impact microbial fitness [17]. After experimentally determining the preferences of the corrinoid-dependent bacteria in our collection, we found that our isolates have considerably lower EC_50_ values for their preferred corrinoids than bacteria for which EC_50_ measurements have previously been reported, indicating lower corrinoid concentrations are required for growth [35, 59, 61, 62]. The EC_50_ values of our isolates are comparable to those of aquatic algae [29, 63], some of which live in environments with corrinoid concentrations in the picomolar range [64] (Fig. 5B and Supplementary Materials Table S1). The ability to use corrinoids at low concentrations could be a useful adaptation to the soil environment where corrinoids may be limiting due to the physical heterogeneity of soil microbial communities, long distances between cells, and fluctuations in water content throughout the year, which make nutrient availability highly variable [13, 65, 66].

All of the dependent isolates were able to use corrinoids that have not been detected in this soil [30]. The concentration of corrinoid chosen for the isolation media was 4-fold higher than the highest EC_50_ and over 16 000-fold higher than the lowest EC_50_ we measured, which explains why isolates were often recovered in their non-preferred corrinoid and why we detected no taxonomic trends in the corrinoid used for isolation. Despite the presence of excess corrinoid in our isolation media, we recovered fewer corrinoid-dependent isolates than expected [26, 50], which may be due to corrinoid-dependent bacteria having additional nutrient dependencies not satisfied by our isolation medium or requiring specific partners for their survival. Indeed, in an analysis of auxotrophies in gut microbiome genomes, most predicted B12 auxotrophs had at least one other vitamin auxotrophy, [6] and among a set of marine isolates, 10/13 B12 auxotrophs were also auxotrophic for at least one other B vitamin [67].

When considering how our classifications of corrinoid metabolism fit into the context of soil microbial ecology, we must consider functional diversity [68]. A contemporary question regarding microbiome function relates to whether groups of microorganisms with shared functions are composed of phylogenetically close organisms or unrelated organisms that share similar metabolic capabilities. Traits such as photosynthesis, methanogenesis, maximum growth rates, and response to soil wet-up tend to be strongly correlated with phylogeny, while others, such as use of specific carbon sources, have weak or no phylogenetic signals [69–71]. Here, we found some phylogenetic trends in corrinoid traits, but overall, the distribution of these traits is patchy across the phylogenetic tree, suggesting that gene loss has occurred at various evolutionary points, possibly due to the frequent emergence of corrinoid dependence and independence [9], or that horizontal gene transfer (HGT) is important in sustaining corrinoid biosynthesis and use. Indeed, corrinoid uptake genes in human gut *Bacteroidetes* are commonly found on mobile genetic elements [72], and *Salmonella typhimurium* and *Lactobacillus reuteri* biosynthesis genes are thought to have been acquired by HGT [73, 74]. The evolutionary history of corrinoids should be explored further to identify which processes have impacted the biosynthesis and use of these cofactors. Using our isolate collection, we were able to carry out the crucial step of validating some genus-level predictions because seven of the genera for which we predicted a genotype were represented among the isolates (Fig. 4).

The characterization of this isolate collection provides key insights about corrinoid-based microbial interactions in soil. We found that dependent isolates were all able to use B12, with most preferring it, and the producers we characterized all synthesize B12 under laboratory conditions, indicating compatibility between corrinoid production and preferences of the dependents. Because oxygen availability is highly variable in soil, and corrinoids other than B12 and ADE are found primarily in anoxic environments, it is possible that these producers synthesize other corrinoids under anoxic conditions. However, the observation that B12 is the major corrinoid found in this soil [30] suggests that most corrinoid producers synthesize B12 in their native environment. Among the producers, however, only some release corrinoid into culture supernatants, suggesting that the corrinoid provider role is fulfilled by a distinct subset of producers [23]. Based on our observation that these providers release corrinoids at levels sufficient to support many dependents in laboratory cultures, we speculate that a small fraction of the community disproportionately provides corrinoids to the dependents. In a similar vein, we previously found that amino acid auxotrophs can be supported by producers at a ratio of over 40:1 [75]. Because corrinoid release cannot be predicted from genomes, it is necessary to combine genotypic predictions of corrinoid production with phenotypic characterizations when studying interactions. This collection of isolates assembled from the same study site will enable further investigation of corrinoid-based interactions via culture-based studies. For example, the mechanisms of corrinoid release, partner specificity in interactions, and competition for corrinoids among dependent isolates can be explored. Focusing on corrinoids simplifies community interactions to only one nutrient and does not take into account other possible interactions that are prevalent in the soil environment, including those involving other shared nutrients [76, 77] or cross-domain interactions [49], which may be affected by environmental fluctuations in the native environment [65]. Nonetheless, focusing on this important class of shared nutrients enabled us to study the diversity of metabolic capabilities that may be prototypical of interactions among soil bacteria and provides a framework to expand the study of other nutrient-sharing interactions.

## Supplementary Material

Alvarez-Aponte_Supplemental_Materials_wrae068

Supp1_VL60_recipe_wrae068

Supp2_bioassay_validation_data_wrae068

Supp3_sequences_used_in_genus_tree_wrae068

Supp4_isolate_metadata_edited_wrae068

Supp5_genus_based_predictions_wrae068

Supp6_corrinoid_needed_vs_provided_wrae068

Supp7_Dose_Response_Fit_Data_wrae068

## Data Availability

The sequencing data generated and analyzed during the current study are available in the NCBI GenBank repository under accession numbers OR878823-OR878983. Code generated during the current study is available from DOI: 10.5281/zenodo.10815172.
