# Supplementary material for: Phylogenetic distribution and experimental characterization of corrinoid production and dependence in soil bacterial isolates
Source: ISME J. 2024 Apr 22;18(1):wrae068. doi: 10.1093/ismejo/wrae068 (PMC11287214; doi:10.1093/ismejo/wrae068)
Supplement: Alvarez-Aponte_Supplemental_Materials_wrae068 [file alvarez-aponte_supplemental_materials_wrae068.pdf]

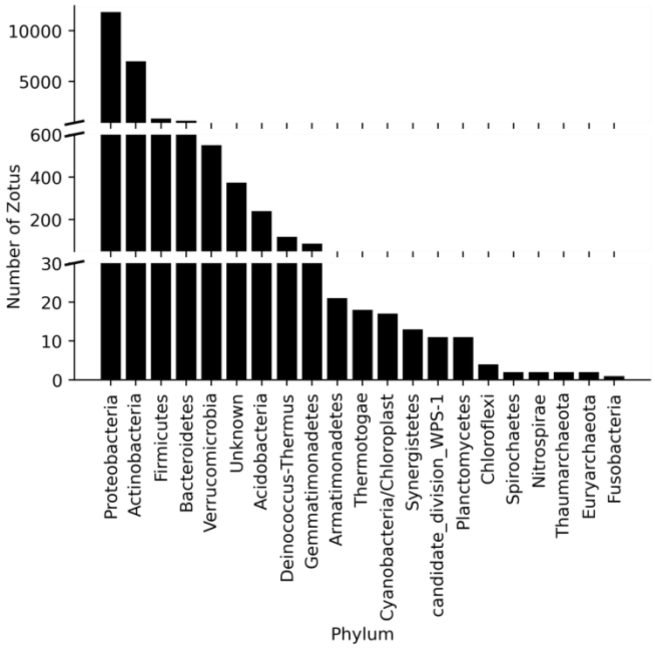

Figure S1. Phylum classification of microbes cultured by the limiting dilution method. Data are shown from Illumina sequencing of amplicons of the 16S V4-V5 region in the second isolation batch. 5.8% of these zOTUs were found to be in clonal wells, while the majority of wells contained two or more zOTUs.

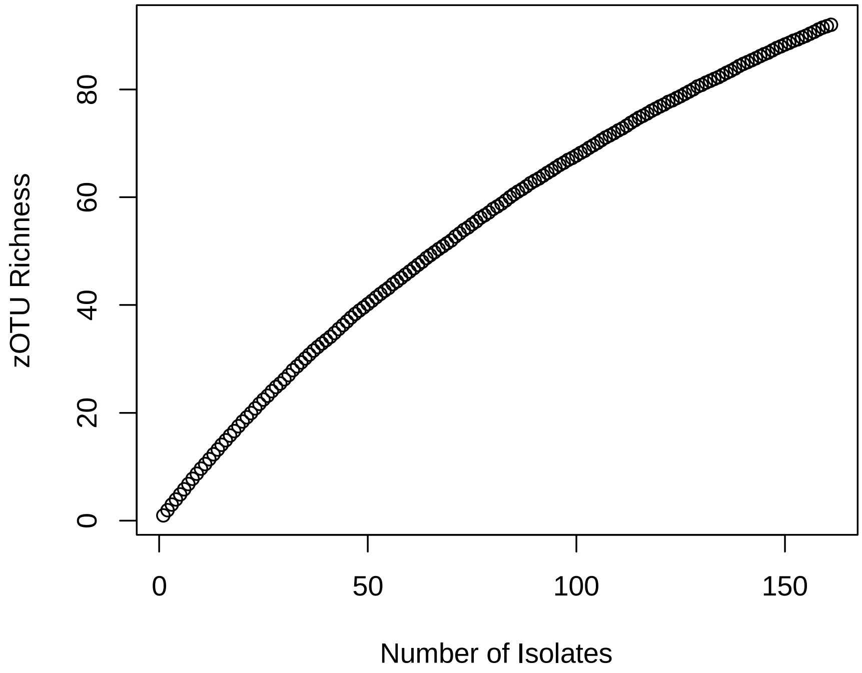

Figure S2. The collector's curve for the collection of 161 isolates shows that our isolation effort was far from saturating the bacterial diversity of this soil.

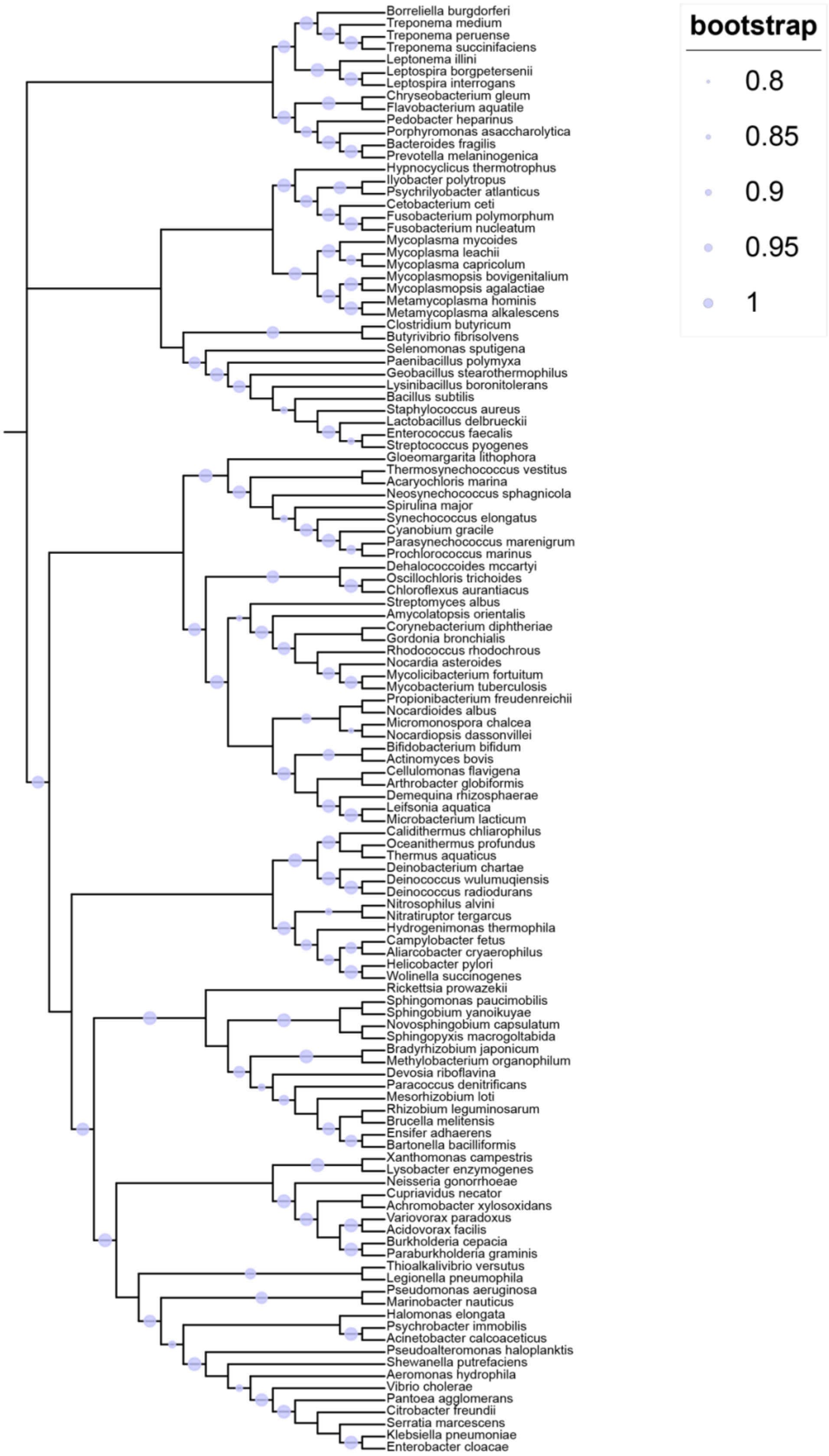

Figure S3. Phylogenetic tree of all type species used to generate the pruned tree in figure 4.

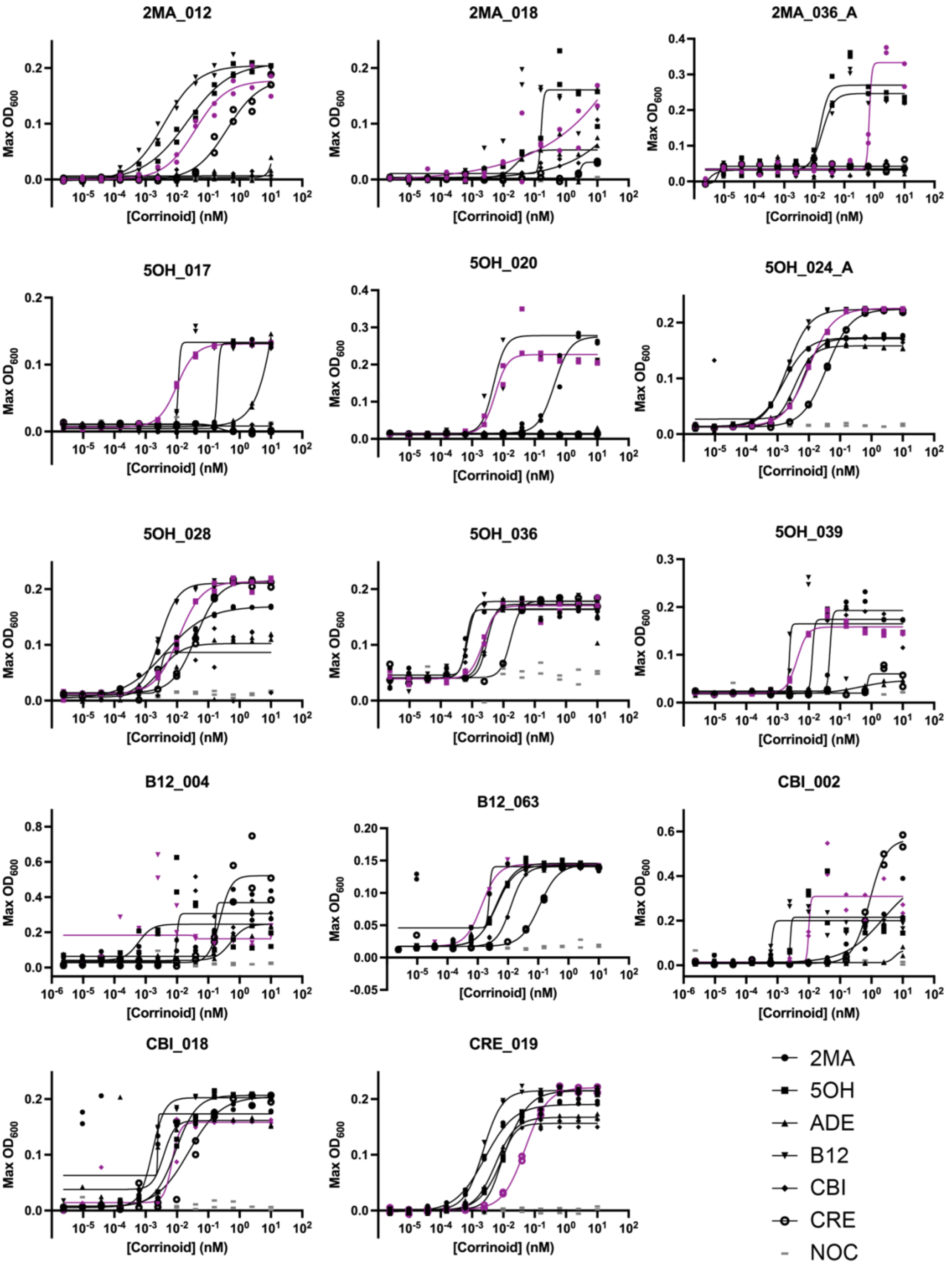
2MA_012
2MA_018
2MA_036_A
5OH_017
5OH_020
5OH_024_A
5OH_028
5OH_036
5OH_039
B12_004
B12_063
CBI_002
CBI_018
CRE_019
Max OD600
[Corrinoid] (nM)
2MA
5OH
ADE
B12
CBI
CRE
NOC

Figure S4. Corrinoid dose-response curves for all tested dependents reveal widespread preferences for B12. The corrinoid used for isolation is shown in purple, and the no corrinoid condition is shown in gray. EC_{50} values calculated from these curves are shown in Figure 5B.

Table S1. Reported EC_{50} values for B12 in bacteria and aquatic eukaryotic algae.

**Domain**	**Organism**	**EC_{50}**	**Reference**
Bacteria	*Akkermansia muciniphila*	56.1 pM	(48)
Bacteria	*Escherichia coli ∆metE*	~0.1 nM	(35)
Bacteria	*Bacteroides thetaiotaomicron*	< 0.4 nM	(66)
Bacteria	*Clostridium difficile*	~1 nM	(68)
Eukarya	B12 dependent mutant of *Chlamydomonas reinhardtii*	28 pM	(29)
Eukarya	*Karenia mikimotoi*	13.1 pM	Tang 2010 PNAS
Eukarya	*Aureococcus anophagefferens*	3.49 pM	(69)
Eukarya	*Rhodomonas salina*	0.36 pM	(69)
Eukarya	*Fibrocapsa japonica*	0.28 pM	(69)
Eukarya	*Chattonella marina*	0.19 pM	(69)
Eukarya	*Prorocentrum minimum*	0.02 pM	(69)
Eukarya	*Pavlova lutheri*	~ 18 pM	(29)
Eukarya	*Ostreococcus tauri*	< 70 pM	(29)
Eukarya	*Amphidinium carterae*	< 70 pM	(29)
Eukarya	*Thalassiosira pseudonana*	< 70 pM	(29)
Eukarya	*Aureococcus anophagefferens*	< 70 pM	(29)
Eukarya	*Lobomonas rostrata*	< 70 pM	(29)
Eukarya	*Euglena gracilis*	< 70 pM	(29)
